# Lateral position for difficult intubation in a patient with history of hemiglossectomy and flap reconstruction: a case report

**DOI:** 10.1186/s40981-022-00509-4

**Published:** 2022-03-03

**Authors:** Fumiko Yokogawa, Katsunori Oe, Maiko Hosokawa, Kenichi Masui

**Affiliations:** 1 Department of Anesthesiology, Showa University School of Medicine, Tokyo, Japan; 2 Department of Anesthesiology, Yokohama City University School of Medicine, Yokohama, Japan

**Keywords:** Difficult airway, Difficult intubation, Laryngoscopy, Fiberoptic intubation, Lateral position, Hemiglossectomy, Reconstructive head and neck surgery, Obstructive sleep apnea

## Abstract

**Background:**

Reconstructive head and neck surgery can alter upper airway anatomy. We report a difficult intubation in a patient with a history of hemiglossectomy and reconstruction.

**Case presentation:**

A 65-year-old female patient, who had undergone hemiglossectomy with the flap reconstruction, underwent video-assisted thoracoscopic esophagectomy for esophageal cancer. After the loss of consciousness during anesthesia induction, we failed to perform direct and oral fiberoptic intubation using a video laryngoscope and nasal fiberoptic intubation without or with video laryngoscope assistance in the supine position. Finally, shifting the patient to the left-lateral position allowed successful nasal fiberoptic intubation. Postoperatively, we were informed that she was unable to sleep in the supine position because of airway obstruction and therefore always slept on her side.

**Conclusion:**

Preanesthetic evaluation of the influence of body position on the airway patency during sleep or sedation may aid in airway management.

## Background

Reconstructive head and neck surgery can change the upper airway anatomy, potentially resulting in difficult intubation [1]. We experienced difficult intubation in a patient with a history of hemiglossectomy and reconstruction. Nasal fiberoptic intubation was completed in the lateral position after unsuccessful nasal fiberoptic intubation in the supine position.

## Case presentation

**Fig. 1:**
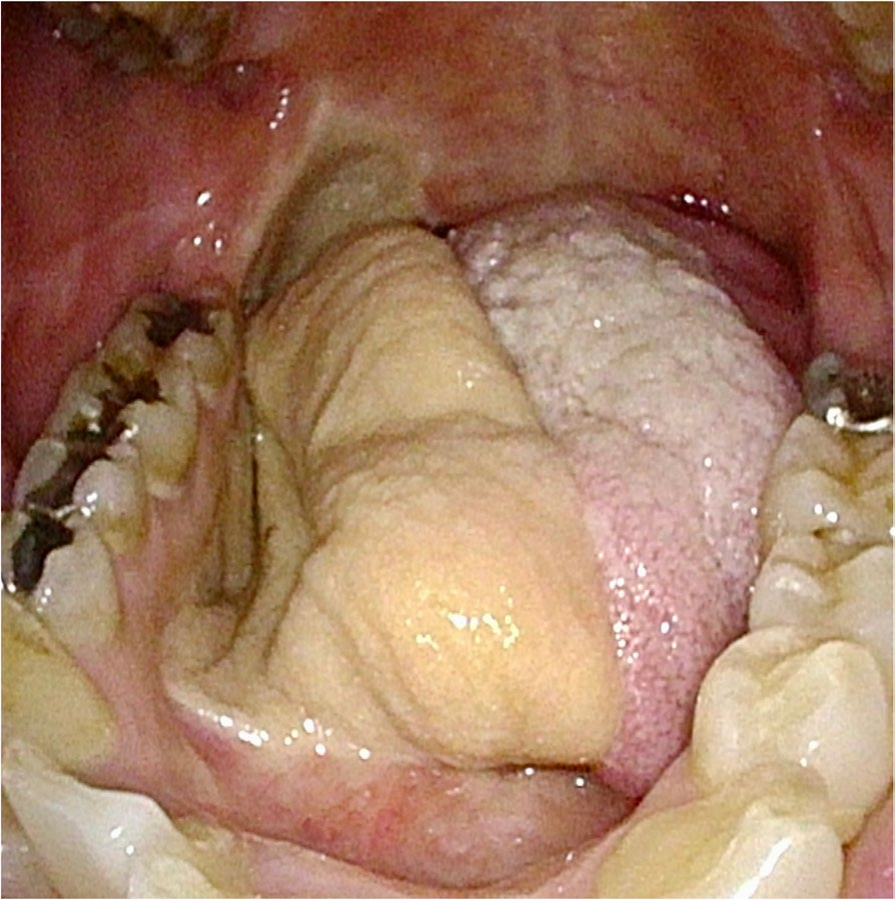
Reconstructed tongue after right-hemiglossectomy using a femoral skin flap

**Fig. 2:**
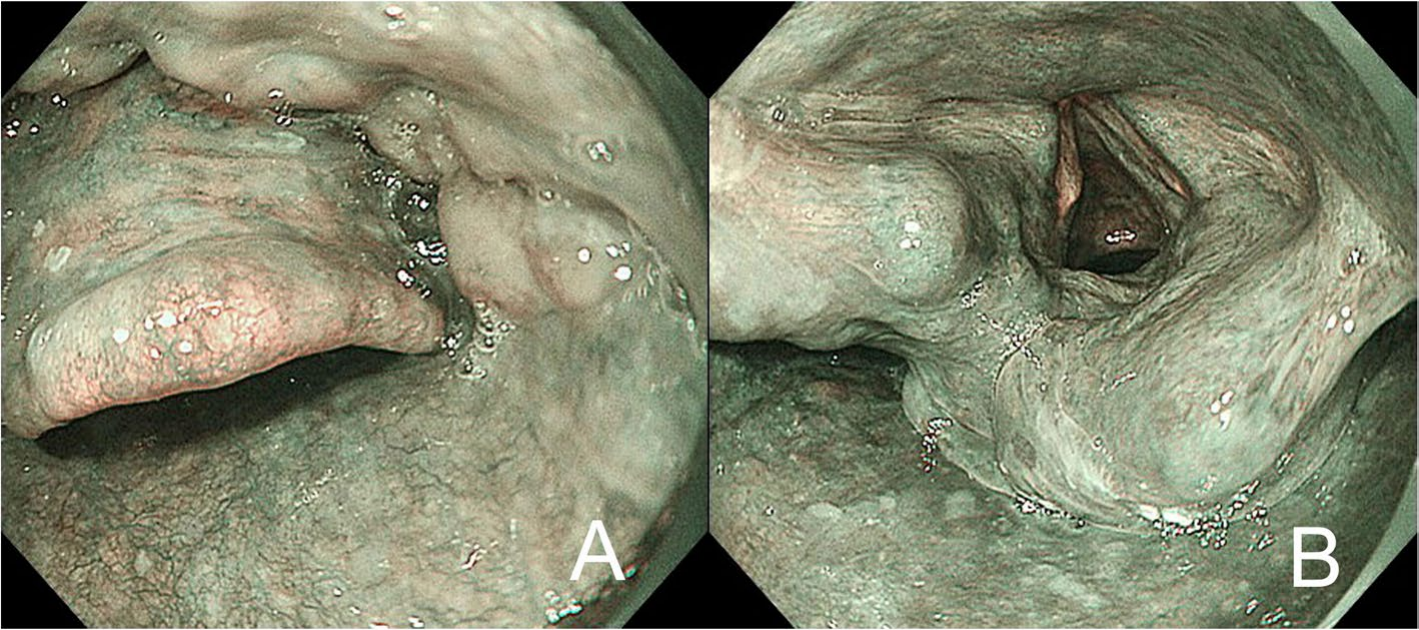
Epiglottis, epiglottis vallecula (**A**), and glottis (**B**) in the lateral position during preoperative gastrointestinal endoscopy under sedation. The protrusion on the left side of the glottis is the corniculate tubercle

**Fig. 3:**
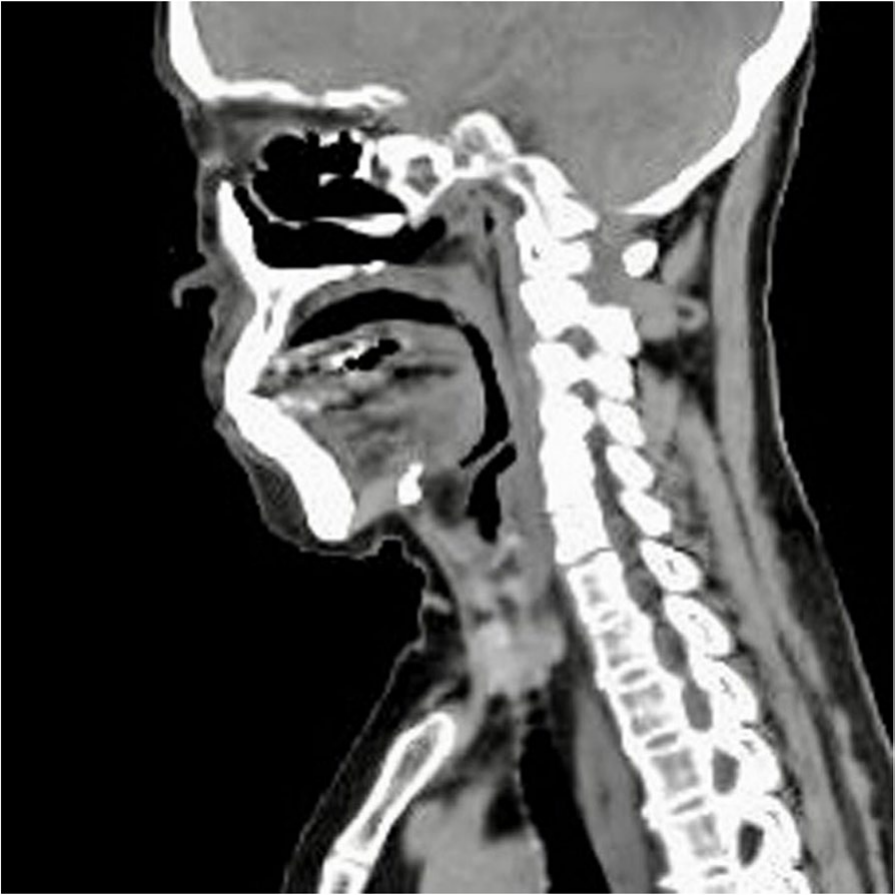
Sagittal computed tomographic view of the upper airway

A 65-year-old woman (height: 149 cm, body weight: 48kg) was scheduled to undergo a video-assisted thoracoscopic esophagectomy for esophageal cancer. She had been diagnosed with tongue cancer and had undergone right hemiglossectomy with flap reconstruction of the tongue using a femoral skin flap 9 years previously at another hospital (Fig. 1). At the preoperative anesthesia visit, the difficult airway and difficult ventilation risks were assessed. During the first minutes, the patient laid in the supine position without respiratory distress. We confirmed that she could lie in the supine position for tens of minutes while awake. Her mouth opening or neck extension were not limited, with Mallampati class II and upper lip bite test class I [2, 3]. Preoperative images during gastrointestinal endoscopy (Fig. 2) and computed tomography (Fig. 3) revealed that no anatomical anomalies or changes were found in the glottis, epiglottis, or epiglottis vallecula.

After gathering preoperative information on airway management, we predicted difficult intubation without difficult mask ventilation and no increased risk of aspiration, increased risk of rapid desaturation, or suspected emergency invasive airway. Therefore, we did not plan awake intubation. The airway management was planned as follows: (1) anesthesia induction with neuromuscular blockade; (2) intubation using a McGrath® video laryngoscope (Aircraft Medical, Edinburgh, Scotland) with a size 4 blade because the estimated distance from the teeth to the vallecula was >11 cm, followed by fiberoptic intubation with or without Airway Scope® (AWS; Pentax, Tokyo, Japan). Moreover, the surgeon found no anomalies or changes in the pharynx during the preoperative gastrointestinal endoscopy before anesthesia.

Oxygen was administered at a flow rate of 6 L/min. Then, a continuous infusion of remifentanil at 0.3 μg/kg/min was started with a bolus of 100 μg fentanyl. After the patient experienced a floating sensation, 80 mg of propofol was administered. As mask ventilation was successfully performed, a bolus of 50 mg of rocuronium was administered. Subsequently, we performed video-laryngoscopy. Poor mobility of the reconstructed tongue did not allow pushing the tongue aside using the McGrath® blade. Because the Cormack and Lehane laryngeal view remained at grade 4 with the backward–upward–rightward pressure maneuver, we terminated the first attempt of laryngoscopy. Desflurane 2% in 100% oxygen was administered and the remifentanil infusion rate was changed to 0.2 μg/kg/min. In the second and third attempts, we tried fiberoptic intubation using the AWS. However, we failed to find the epiglottis and esophagus using fiberoptic bronchoscopy. On the fourth attempt, a fiberoptic bronchoscopy was inserted into the left nasal cavity through a tracheal tube. We were unable to insert the bronchoscopy into the hypopharynx because we could not move the bronchoscope forward. Additionally, the McGrath® was inserted into the oral cavity. However, we only identified edematous mucous membranes in the larynx and were unable to locate the epiglottis. As the peripheral oxygen saturation decreased, we interrupted the intubation procedure and performed two-person mask ventilation. The lowest peripheral oxygen saturation level was 92%. Meanwhile, the surgeons advised that they could observe both the glottis and epiglottis during gastrointestinal endoscopy in the left-lateral position. The patient was then moved from the supine to the left-lateral position. Fiberoptic intubation via the nasal cavity was attempted again. Although an edematous membrane was observed in the larynx, a narrow gap was observed on the distal side of the epiglottis-like edematous structure. After moving the fiberoptic bronchoscope into the gap, the normal glottis, ring-shaped tracheal cartilage, and tracheal bifurcation were finally identified. The tracheal tube was smoothly intubated using a bronchoscopy guide. Post-surgery, the patient was admitted to the intensive care unit under sedation and mechanical ventilation with tracheal intubation.

The following morning, the patient’s trachea was carefully extubated using the following procedure: (1) the patient was positioned in the left-lateral position, (2) a fiberoptic bronchoscope was inserted into the tracheal tube, (3) the trachea was extubated with the bronchoscope remaining in the trachea, and (4) the glottis, epiglottis, and epiglottis vallecula were observed using bronchoscopy. During this procedure, no edematous membrane was observed in the pharynx. After extubation, the glottis shifted to the ventral side and upper airway stenosis and obstruction were not observed.

After the patient clearly recovered, we asked her whether she could sleep in the supine position before and after the hemiglossectomy. She answered, “I always sleep in the lateral position. If I sleep in the supine position, I wake up because of airway obstruction. This did not happen before my hemiglossectomy.” She retained no memory from the anesthesia induction.

## Discussion

In this case, we predicted a difficult intubation due to post-hemiglossectomy with flap reconstruction, but not “cannot intubate” situation using fiberoptic bronchoscopy in the supine position. We postoperatively evaluated the “preoperative risk class” for difficult mask ventilation combined with difficult laryngoscopy [4]. She had three risk factors (presence of teeth, age ≥46, and obstructive sleep apnea) among the 12 risk factors for the classification, which determined a low risk, 0.18% of the occurrence rate, of difficult mask ventilation and difficult laryngoscopy. However, both mask ventilation and laryngoscopy in the supine position were difficult in this patient. One-person facemask ventilation with excessive resistance to gas ingress after the third attempt of tracheal intubation, and impossible visualization of any portion of the vocal cords after three plus one attempts at video-laryngoscopy conformed with the criteria indicative of difficult facemask ventilation and difficult laryngoscopy, respectively [2]. The preoperative risk class for difficult mask ventilation combined with difficult laryngoscopy may be unsuitable for predict difficulties in patients with a history of hemiglossectomy with flap reconstruction.

Laryngoscopy was attempted three plus one times in the supine position. For the first attempt, we prepared a McGrath® video laryngoscope with a long blade (size 4). However, the blade length was short for this patient. One reason was the poor mobility of the flap reconstructed tongue. Accordingly, we decided to perform fiberoptic intubation with AWS for the next attempts and then nasal fiberoptic intubation without or with McGrath®. Although we performed a limited number of laryngoscopy within the limits of airway guidelines [2], these procedures produced larynx edema resulting in a difficult airway. Nasal fiberoptic intubation without laryngoscope assistance might have been a better choice from the first to third attempts to avoid laryngeal edema.

The left-lateral position was effective for the successful intubation in this case, facilitating the visualization of the glottis and epiglottis during gastrointestinal endoscopy. Although a randomized controlled trial in patients without known or predicted difficult airway revealed that turning from the supine to the left-lateral position hinders the laryngoscopic view [5], and seemed inappropriate in such patients, another study clarified that the lateral position decreases the collapsibility of the passive pharynx in patients with obstructive sleep apnea [6]. The lateral position in obstructive sleep apnea during anesthesia induction would be a better choice for patients with difficult mask ventilation and for difficult laryngoscopy. In this case, a postoperative interview revealed that the supine position caused airway obstruction during sleep, but not while awake. During the preoperative anesthesia round, the patient was placed in the supine position without respiratory distress. Therefore, we were unaware of the risk of difficult intubation in the supine position. Preoperative gastrointestinal endoscopy was performed under sedation and spontaneous respiration, without airway obstruction. During the preoperative interview in patients who have undergone a hemiglossectomy with flap reconstruction or other surgeries reducing the airway patency with obstructive sleep apnea, detailed information on airway patency, including the influence of body position during sleep or sedation, may help determine the airway management during general anesthesia.

Awake intubation might have been an alternative strategy for airway management in this patient. The 2020 practice guidelines for difficult airway management recommend awake intubation in patients with suspected difficult laryngoscopy combined with (1) suspected difficult ventilation with face mask/supraglottic airway, (2) significantly increased risk of aspiration, (3) increased risk of rapid desaturation, or (4) suspected difficult emergency invasive airway [2]. Although this patient was suspected of having difficult laryngoscopy, we predicted none of the four conditions. Accordingly, tracheal intubation was attempted under general anesthesia.

In conclusion, we experienced difficult mask ventilation and video-laryngoscopy in a patient who had undergone a hemiglossectomy with the flap reconstruction 9 years prior. We failed to perform video-laryngoscopy using McGrath®, fiberoptic intubation with AWS, and nasal fiberoptic intubation without or with McGrath® assistance in the supine position. Finally, the left-lateral position facilitated successful nasal fiberoptic intubation. This patient always slept in the lateral position due to obstructive sleep apnea in the supine position after hemiglossectomy. The lateral position may facilitate successful laryngoscopy in a “difficult laryngoscopy” situation in patients with a history of hemiglossectomy with the flap reconstruction or surgery, which reduces the airway patency concurrently with obstructive sleep apnea. Preanesthetic interviews to assess airway patency to determine the influence of body position during sleep or sedation may help determine an appropriate strategy of airway management.

## Data Availability

Not applicable.

## Abbreviation

AWS: Airway scope
